# P04-11 A physical activity intervention designed to improve multi-dimensional health and functional capacity of frail older adults in residential care: a randomised controlled feasibility study

**DOI:** 10.1093/eurpub/ckac095.065

**Published:** 2022-08-29

**Authors:** Bridgitte Swales, Anna Whittaker, Gemma Ryde

**Affiliations:** Faculty of Health Sciences and Sport, University of Stirling, Stirling, United Kingdom

**Keywords:** Ageing, strength, frailty, well-being, feasibility

## Abstract

**Background:**

Frailty is a common and clinically significant multi-dimensional syndrome associated with adverse health outcomes such as hospitalisation, disability, and mortality among older adults. Physical activity interventions have been shown to be effective in the prevention and treatment of functional decline and frailty, with strength training protocols reporting improvements in mobility, balance, preservation of muscle function and independence. Due to the trial's exploratory and feasibility design a small sample was used to investigate the acceptability and practicality in long term care. This includes addressing the HEPA priority of improving the health and well-being of people with chronic conditions and/or frailty.

**Methods:**

Eleven older adults (aged >65 years) were randomised to the intervention or wait-list control. A 6-week strength training protocol for 35 mins 3x/week used resistance machines, specially designed for older adults, in a residential care home. Mixed methods were used to assess the feasibility and acceptability of the intervention and research measures and indicate meaningful differences in outcomes. Feasibility was measured through adherence statistics and focus groups/interviews with staff and participants. Pre- and post-intervention levels are given as descriptive statistics for physiological, psychosocial, cognitive, and functional measures. Intervention effect is illustrated through mean difference (95% Confidence Intervals) pre- to post-intervention in the intervention group.

**Results:**

Intervention group (n = 6) adherence was 98.9%. Interviews revealed participants and staff found the measures and intervention acceptable, practicable and beneficial. Mean differences pre- to post-intervention indicated meaningful clinical difference in measures of strength, functional capacity and frailty. Psychosocial variables (stress, depression, social support), immunological measures, cognition and Activities of Daily Living Scores did not show meaningful change.

**Conclusion:**

A strength training intervention protocol for frail care home residents was beneficial, with findings supporting a future randomised controlled trial. High adherence and clinically meaningful differences for frailty and physical function support the use of such interventions to improve multi-dimensional health, maintain functional capacity and independence, and enhance social participation in frail older adults. This study and further research may help inform practical physical activity initiatives for frail older adults, and support strategies prioritising health and well-being, and the prevention of functional decline and frailty.

